# Non-cytotoxic *Thymus capitata* extracts prevent Bovine herpesvirus-1 infection in cell cultures

**DOI:** 10.1186/s12917-014-0231-6

**Published:** 2014-09-27

**Authors:** Ramzi Boubaker–Elandalousi, Marwa Mekni–Toujani, Belhassen Kaabi, Imen Larbi, Mohamed-Fethi Diouani, Mohamed Gharbi, Hafidh Akkari, Fatma B’chir, Abdeljelil Ghram

**Affiliations:** Université Tunis El Manar, Laboratoire d’Epidémiologie et Microbiologie Vétérinaire, Institut Pasteur de Tunis, Place Pasteur BP 74, 1002 Tunis, Tunisia; Université de la Manouba, Institut Supérieur de Biotechnologie, BiotechPôle, BP–66, 2020 Sidi Thabet, Tunisia; Université de la Manouba, Laboratoire de Parasitologie, École Nationale de Médecine Vétérinaire de Sidi Thabet, 2020 Sidi Thabet, Tunisia; Université de la Manouba, Laboratoire des Substances Naturelles, Institut National de Recherche et d’Analyse Physico–chimique – Pôle Technologique Sidi Thabet, 2020 Sidi Thabet, Tunisia

**Keywords:** *Thymus capitata*, Cytotoxicity, Antiviral, Madin-Darby Bovine Kidney cell, *Bovine Herpesvirus type1*

## Abstract

**Background:**

Bovine herpes virus type 1 (BHV-1) still causes great economic loss to the livestock industry and trade because there aren’t any available drugs that proved to be fully effective against it. In this study, the cytotoxicity and the antiviral activities of the *Thymus capitata* extracts were evaluated for the development of new, non toxic and specific anti-herpesvirus drug. Aqueous extracts (AE), ethanolic extracts (EE) and essential oil (EO) of the aerial parts of *Thymus capitata* were analyzed to determine their chemical compositions by gas chromatography, and high performance liquid chromatography combined with mass spectrometry. Their cytotoxicity and antiviral activities against Bovine Herpesvirus type 1 (BHV-1) were evaluated by quantifying the reduction of the viral cytopathic effect using Madin-Darby Bovine Kidney cell line with colorimetric assay. *T. capitata* extracts were added at different stages of the viral infection to investigate and better quantify their potential inhibitory effects.

**Results:**

Polyphenols and flavonoids were the major compounds found in *T. capitata* EO, EE and AE. The cytotoxic concentrations at 50% were 48.70, 189 and 289 μg ml^−1^ for EO, EE and AE, respectively. The inhibitor concentrations at 50% for the EO, EE and AE, were 3.36, 47.80 and 164 μg ml^−1^, respectively. The selectivity index anti-BHV-1 values were 14.49, 3.95 and 1.81 for EO, EE and AE, respectively. Thus, the EO extracts were the most efficient antiviral compounds. *T. capitata* extracts affect mainly the adsorption of BHV-1 virus to host cells.

**Conclusion:**

*T. capitata* extracts inhibit the viral replication by interfering with the early stages of viral adsorption and replication. Thus, *T. capitata* is a potential candidate for anti-herpesvirus treatment.

## Background

Bovine herpesvirus type 1 (BHV-1), a member of the subfamily *Alphaherpesvirinae*, is a virulent pathogen causing significant economic losses to the livestock industry, worldwide [1]. The virus is associated with a variety of symptoms including rhinotracheitis, vulvovaginitis, balanoposthitis, abortions, conjunctivitis and generalized systemic infections [2]. Infectious bovine rhinotracheitis (IBR), a wide spread enzootic herpetic infection caused by BHV-1, is classified in the list B of diseases by the Office International des Epizooties [2]. BHV-1-induced immunosuppression leads frequently to secondary bacterial infections. Thus, BHV-1 is an important cofactor in the bovine respiratory disease complex which has great financial impacts [1,2]. Furthermore, BHV-1 has received increasing attention as a surrogate model for anti-herpes virus drug screenings. On the other hand, plant-derived antiviral extracts are of great interest for the development of new, non-toxic, more effective and specific anti-herpesvirus active molecules. Indeed, several trials using plant extracts have shown *in vitro* anti-BHV-1 activities at early and/or late stages of the viral replication, such as: *Phyllanthus orbicularis* [3], *Erythroxylum deciduum*, *Lacistema hasslerianum* (chodat), *Xylopia aromatica* [4], *Heteropteris aphrodisiaca* [5], *Acacia nilotica* (gum arabic tree) [6], *Lippia graveolens* (Mexican oregano or redbrush lippia) [7], *Guettarda angelica* (Velvetseed) [8], *Prunus myrtifolia* (West Indian cherry), *Symphyopappus compressus* [9], and *Pimpinella anisum* (Anise) [10].

A large number of plant extracts from *Lamiaceae* were also examined for their potential antiviral activity against herpesvirus, such as: *Melissa officinalis* (lemon balm), *Mentha piperita* (pepper-mint), *Prunella vulgaris* (prunella), *Rosmarinus officinalis* (rosemary), *Salvia officinalis* (sage) and *Thymus vulgaris* (thyme) [11–14]. Besides, different thyme species have been screened for antibacterial, anthelmintic, antifungal and antioxidant activities, and as immune modulator [15,16]. However, to the best of our knowledge, cytotoxic and antiviral activities of different *T. capitata* (Order of *Lamiales*, Family *of Lamiaceae*) extracts against BHV-1 have never been tested.

In the present study, we have determined the chemical compositions and the cytotoxicity effects of different extracts of the *T. capitata* areal parts, collected in Matmata region (Southern Tunisia). The aqueous (AE) and ethanolic extracts (EE) and the essential oil (EO) were tested, since significant differences in antimicrobial activities between these extracts have been previously reported [11,12].

## Methods

### Collection and preparation of the plant materials

Fresh *T. capitata* plants were collected in June 2011 from Matmata locality in the South East of Tunisia (33°32′ North 9°58′ East). Aerial parts of the plants (leaves, stems and flowers) were separated, thoroughly rinsed in running tap water and air dried at room temperature during 14 days, then pulverized, grounded to fine powder and stored at +4°C until use.

### Preparation of the extracts

The AE and EE were prepared as previously described by Boubaker Elandalousi *et al.* [16]. The EO was prepared by dissolving 100 g of dried plant material in 1 liter of distilled water, and then submitted to microwave-assisted hydro-distillation at +40°C during 4 hours, in a Clevenger-type apparatus.

Stock solution (10 mg ml^−1^) of EO and EE were dissolved in Dulbecco’s modified Eagle’s cell culture medium (DMEM) with 0.5% dimethylsulfoxide (DMSO). All extracts were sterilized by filtration (0.22 mm filter), dried and kept in dark flask at +4°C until tested.

### Analyses of *T. capitata* EO, EE and AE compositions

#### EO and EE gas chromatography/mass spectrometry (GC/MS) analysis

The GC-MS unit consists on a Perkin-Elmer Autosystem XL gas chromatograph, equipped with HP-5MS fused-5% Phenyl Methyl Siloxane capillary column (Agilent, 30 m×0.25 mm, film thickness 0.25 μm). It is interfaced with Perkin-Elmer Turbo mass spectrometer at specific operating conditions (injector temperature: 250°C; carrier gas: Helium adjusted to a linear velocity of 37 cm s^−1^; flow rate: 1 ml min^−1^; volume of injected sample: 1 μl; split ratio: 50:1; ionization energy: 70 Ev; ion source temperature: 200°C; scan mass range m z^−1^: 50–550 and interface line temperature: 300°C).

#### AE high performance liquid chromatography/mass spectrometry (HPLC/MS) analysis

HPLC-MS (Agilent, Waldbronn, Germany) separation was performed using an Agilent C18 reverse-phase column (150×4.6 mm) which was maintained at 33°C, with a direct injection of 25 μl of the extract at a 100 bars pressure and a flow rate of 0.25 ml min^−1^. Elution was performed by gradient mode, using water and acetic acid (999/1 v/v), and acetonitrile in mobile phases. The gradient program was set to 5% for the 5 first minutes, increased linearly to 100% for 65 minutes, remained at 100% for three minutes and decrease to 5% during the last 69 minutes. Chromatographic peaks were investigated with Mass Lynx Software (Waldbronn, Germany).

The Mass spectroscopy (MS) was performed using a Micro mass Quattro Ultima PT MS model (Waldbronn, Germany) at the following operating conditions: capillary voltage (3.20 kV); capillary temperature (300°C); multiplier (550 V) and cone gas flow (60 L Hr^−1^). The ion trap detector with electro-spray ionization source was used for quantification, and it was set in negative ionization mode.

Extracts constituents were identified by comparing their mass spectra or retention indices as HPLC-MS spectra with those of reference chemical compounds gathered from the *Institut National de Recherches et d’Analyses Physico-chimiques*, Tunisia - LMS library, and commercially available standards from published libraries.

### Cell line

Monolayer cultures of MDBK cell line were grown in DMEM supplemented with 10% fetal calf serum, 100 IU ml^−1^ penicillin G and 100 mg ml^−1^ streptomycin. Two hundreds microliters of 1×10^6^ ml^−1^ cell suspension were put into each well of a 96-well culture plate, for both cytotoxicity and antiviral assays. All plates were maintained in an incubator at 37°C and 5% CO_2_ atmosphere.

### Virus

Stock virus of the Cooper-1 (Colorado-1) strain of BHV-1, obtained from the American Type Culture Collection (Rockville, MD, USA), was propagated in MDBK cell cultures. The infected cell supernatant fluids were harvested, titrated and stored at −80°C until use.

For the virus titration, MDBK cells were seeded in 24-well culture plate and incubated at 37°C. After 24 hours, serial dilutions of the virus stock were prepared in culture medium, and each dilution was added to 4 wells. After an additional 72 hours of incubation, the cytopathic effect (CPE) in each well was recorded. The viral Titer of BHV-1 was calculated by Reed and Muench method, as described previously [17].

### Cytotoxicity assays

To examine the effect of *T. capitata* extracts on the growth and the viability of the cultured cells, serial dilutions of extracts (from 5 to 500 μg ml^−1^) were prepared in DMEM, in triplicate, and 200 μl of each dilution was added to the 96-well culture plates. The maximum non-toxic concentration (MNTC) was determined by cell morphology alterations, estimated under a light microscope at ×100 magnifications during the 3 days incubation at 37°C. Monolayer cells incubated with only DMEM were used as cell controls. The cells were fixed with 1% glutaraldehyde for 10 min, stained with 0.1% crystal violet during 30 min, and the optical density was determined by a spectrophotometer at 620 nm wavelength. The cell viability was calculated using the following formula:

$$ \mathrm{Cell}\ \mathrm{viability}\left(\%\right)=100\times \left(\mathrm{Ab}{\mathrm{s}}_{\mathrm{s}\mathrm{ample}}\hbox{--} \mathrm{Ab}{\mathrm{s}}_{\mathrm{cell}\ \mathrm{free}\ \mathrm{blank}}\right)/\mathrm{Ab}{\mathrm{s}}_{\mathrm{mean}\ \mathrm{media}\ \mathrm{control}} $$

Abs: the absorbance

The 50% cytotoxic concentration (CC_50_) was calculated as the concentration causing 50% cell death.

### Antiviral assays

Only non cytotoxic and antiviral concentrations of each plant extract below the MNTC (EO: 2, 3, 4 and 5 μg ml^−1^; EE: 50, 55 and 60 μg ml^−1^ and AE: 160, 170 and 180 μg ml^−1^) were tested to assess anti-BHV-1 activity.

To elucidate the mode of antiviral action, the MDBK cells were incubated with various plant extracts for 3 days, following 3 scenari: before and after viral infection, simultaneous infection.

#### Cell culture pretreatment (before infection)

Pre-treatment of cell cultures was performed by exposing the cell monolayers to different concentrations of the test compounds in maintenance medium (200 μl) for 2 hours at 37°C. After treatment, the cell monolayers were washed thoroughly with phosphate buffered saline (PBS), infected with 200 μl BHV-1 suspension at 10^3^ Tissue Culture Infective Dose 50% (TCID_50_) ml^−1^ of virus and observed for viral cytopathic activity during 72 hours incubation.

#### Inhibition of virus attachment (simultaneous infection) assay

BHV-1 suspension at a viral concentration of 10^3^TCID_50_ ml^−1^ of virus was mixed (v/v: 100 μl) with different concentrations of the test extracts at room temperature. Then, cells were incubated immediately with 200 μl of the mixtures for 72 hours at 37°C and 5% CO_2_ atmosphere.

#### Post-infection assay

Two hundred microliters of viral suspension at 10^3^ TCID_50_ ml^−1^ in culture medium were adsorbed to MDBK cells for 2 hours at 37°C. Cells were then washed with PBS, and the medium was replaced with 200 μl DMEM containing the EO, EE and AE of *T. capitata* at serial dilutions, which were then incubated for 72 hours at 37°C and 5% CO_2_ atmosphere.

The controls consisted of untreated infected cells for virus control, confluent monolayer cells were infected with the virus at 10^3^ TCID_50_ ml^−1^ of virus; treated uninfected cells for plant extract controls; and untreated uninfected cells for cell control. After 3 days of incubation, the optical density (OD) was determined as previously described. The level of antiviral activity of the plant extracts was calculated using the following formula:

$$ \mathrm{Antiviral}\ \mathrm{activity}\ \left(\%\right) = 100 \times \left(\mathrm{Ab}{\mathrm{s}}_{\mathrm{s}\mathrm{ample}}\hbox{--}\ \mathrm{Ab}{\mathrm{s}}_{\mathrm{virus}\ \mathrm{control}}\right)/\left(\mathrm{Ab}{\mathrm{s}}_{\mathrm{cell}\ \mathrm{control}}\hbox{--}\ \mathrm{Ab}{\mathrm{s}}_{\mathrm{virus}\ \mathrm{control}}\right) $$

The absorbance (Abs) of the virus control was calculated by mixing 100 μl ×10^3^ TCID50 of virus suspension with 100 μl of culture medium without plant extracts. The absorbance of cell controls was estimated by mixing 100 μl of MNTC of each extracts with 100 μl of culture medium [18]. The 50% effective antiviral concentration (IC_50_) was calculated as the antiviral concentration causing 50% inhibition of virus-induced CPE. The selectivity index (SI) was calculated as the ratio of CC_50_ and IC_50_.

### Statistical analysis

The data are presented as means ± standard deviation (S.D). The T-test was used to compare differences between mean groups. A correlation test between concentration extracts and viral inhibition was also performed. A p-value < 0.05 was considered to imply statistical significance. The statistical analysis were performed using the R software for statistical computing (v3.01, available from: www.r-project.org), and GraphPad Prism software®, release 3.0.

## Results

The results of the chemical composition and the cytotoxic and antiviral activities of each plant extract are presented in Tables 1, 2, and 3, respectively.

**Table 1:** **List of chemical compounds (descending order) in essential oil and ethanolic extract identified by GC–MS and in aqueous extract identified by HPLC–MS of** ***Thymus capitata***

**Molecule**	**Essential oil (%)**	**Ethanolic extract (%)**	**Aqueous extract**
3-Methyl-4-isopropylphenol: thymol	73.38	71.22	Apigenin-6,8- di C-glucoside
Camphor	ND	17.18	Luteolin-7- rutinoside
Benzene	10.86	6.32	Hesperidin
Caryophyllene	2.55	1.11	Apigenin-7- glucoronide
Linalool	1.97	ND	Eupafolin- glucoside
Caryophyllene oxide	1.87	0.98	Acethyl- luteolin- glucuronide
3-Cyclohexen-1-ol	0.92	0.61	Rosmarinic Acid
3-Cyclohexen-1-ol	0.92	ND	
1,6-Octadien-3-ol	ND	0.83	
Eugenol	0.81	ND	
Borneol	0.78	0.64	
Beta.-Myrcene	0.72	0.15	
Dimethyl Sulfoxide	0.56	ND	
(+)-4-Carene	0.50	ND	
3 Hydroxy-1-octene	0.41	ND	
4,5-epoxy-1-isopropyl-4-methylcyclohexane	ND	0.29	
Phenol	0.28	0.26	
Phenol, 2,3,4,6-tetramethyl	0.22	ND	
3-Octanol	0.20	ND	
1,4-Cyclohexadiene	0.18	ND	
Octahydrophenanthrene	0.14	ND	
2,6,11,15-Tetramethyl-hexadeca-2, 6,8,10,14-pentaene	0.14	ND	
Naphthalene	0.12	ND	
Alpha.-Caryophyllene	0.12	ND	
Endo-Borneol	0.11	ND	
Lopentadiene	0.09	ND	
Trans-Linalool oxide	0.05	ND	
Phenol Carvacrol	0.04	ND	
Acetaminophenol	0.04	ND	
Phenanthrene	0.03	ND	
**Total**	**98.01**	**99.59**	

*ND*: Not detected.

**Table 2:** **Cytotoxic activities of essential oil, ethanolic and aqueous extracts of** ***Thymus capitata***

**Concentration (μg ml** ^**−1**^ **)**	**Mean cell survival percentage ± standard deviation**		
	**Essential oil (n = 3)**	**Ethanolic extract (n = 3)**	**Aqueous extract (n = 3)**
5	100 ± 1.03	95.63 ± 3.31	NA
10	93.50 ± 1.13	93.18 ± 3.87	NA
20	84.51 ± 1.59	96.05 ± 2.87	NA
50	47.42 ± 0.64	92.83 ± 3.27	100 ± 0.74
100	14.77 ± 0.07	79.61 ± 3.64	92.21 ± 2.41
200	2.91 ± 0.03	45.83 ± 1.90	74.20 ± 1.71
300	1.63 ± 0.05	28.97 ± 1.90	48.35 ± 1.21
500	1.21 ± 0.04	26.80 ± 1.47	28.68 ± 0.38

*NA*: Not applicable.

**Table 3:** **Antiviral activities of selected** ***Thymus capitata*** **extracts against bovine herpesvirus type1**

**Extract**	**Yield** ^**a**^ **(%)**	**CC**_**50**_^**b**^ **(μg ml** ^**−1**^ **)**	**IC**_**50**_^**c**^ **(μg ml** ^**−1**^ **)**	**SI** ^**d**^
**Essential oil**	1	48.70	3.36	14.49
**Ethanolic extract**	10.05	189	47.80	3.95
**Aqueous extract**	3.25	298	164	1.81

^a^% w/w = g of extracts 100 g^−1^ of dried and ground plant material.

^b^Cytotoxic concentration 50%; ^c^: inhibitor concentration 50%;

^d^Selectivity index = CC_50_/IC_50._

### *Chemical analysis of* Thymus capitata *EO, EE and AE*

A total number of 28 components were identified in *T. capitata* EO; phenols were the major constituents, mainly the 3-Methyl-4-isopropylphenol: thymol (73.38%). Other compounds were also present but with lower concentrations such as caryophyllene (sesquiterpene, 2.55%), linalool (monoterpene, 1.97%), eugenol (phenol, 0.81%) and borneol (monoterpene, 0.78%). Aromatic hydrocarbons (benzene, naphthalene, anthracen) and terpens (myrecen, caren) were also present in *T. capitata* EO with lower concentrations (Table 1).

Twelve compounds were identified in EE; most of them were phenols such as 3-Methyl-4-isopropylphenol or thymol (71.22%), camphor (17.18%), benzene (6.32%) and caryophyllene (1.11%) (Table 1).

Apigenin and luteolin derivatives were the most abundant phenolic compounds (flavonoid) found in AE, whilst rosmarinic acid was a minor one (Table 1).

Several phenolic components were present with roughly the same concentrations in both EO and EE, mainly 3-Methyl-4-isopropylphenol: thymol (73.38% and 71.22%, respectively) followed by benzene (10.86% and 6.32%, respectively).

### Cytotoxicity

**Figure 1:**
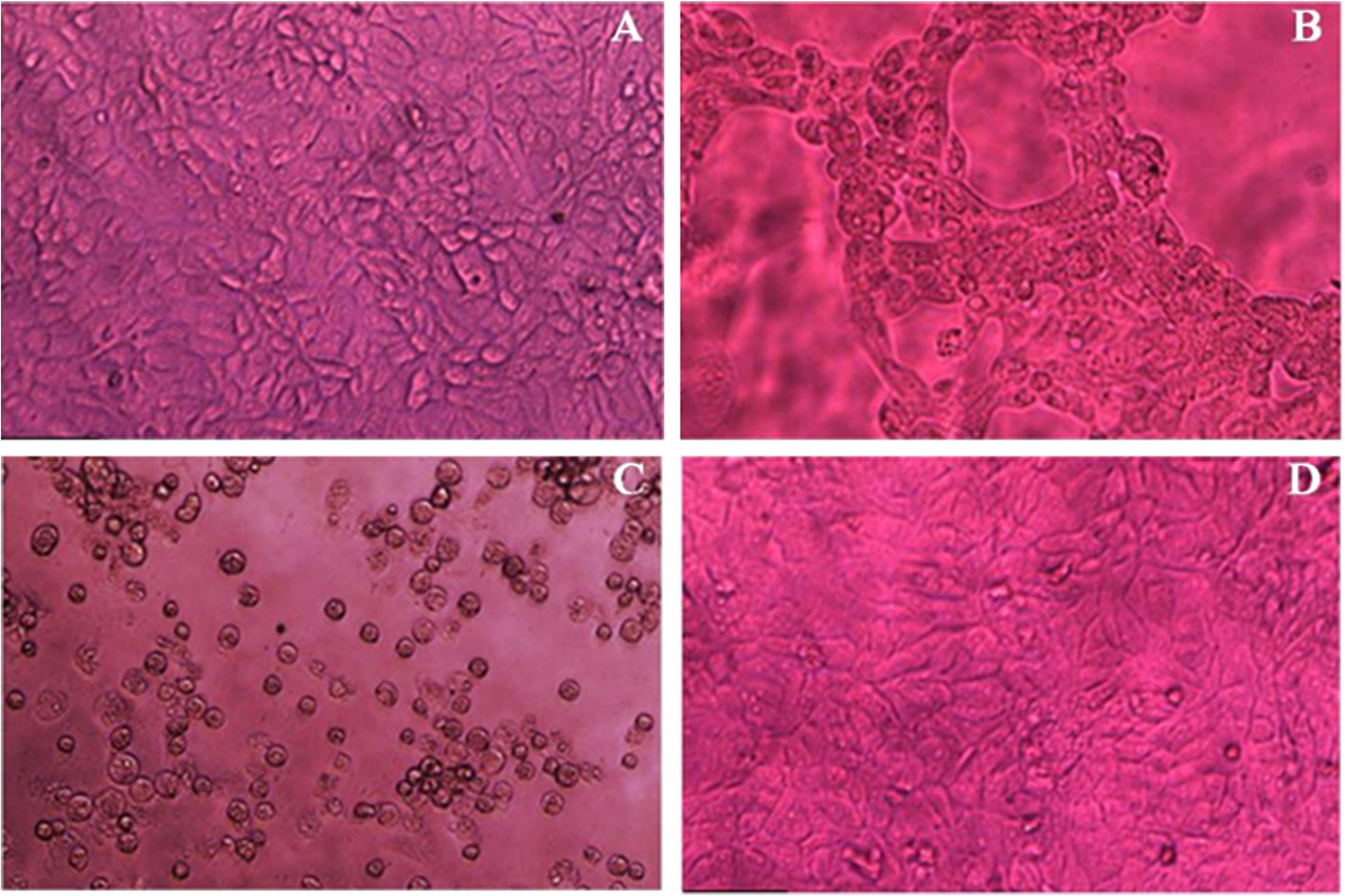
**Cytotoxicity and cytopathic effects on Madin-Darby Bovine Kidney cells (×100) after addition of EO, EE and EA of** ***Thymus capitata*** **before infection with 10** ^**3**^ **TCID**_**50**_ **ml** ^**−1**^ **of Bovine herpesvirus type 1 (BHV-1). A**: Cell control, not infected with BHV-1, after 72 hours of incubation. **B**: Virus control (cytopathic effect), untreated MDBK cell culture, 72 hours after BHV-1 infection with 10^3^ TCID_50_ ml^−1^. **C**: Cytotoxic effect, MDBK treated with extracts of EO (50 μg ml^−1^), 72 hours of incubation. **D**: Viral inhibition, MDBK cells were pre-treated with EO (5 μg ml^−1^) for 2 h, then infected with BHV-1 at 10^3^ TCID_50_ ml^−1^.

To examine the effect *T. capitata* extracts on the growth and the viability of cultured cells, EO, EE and AE were serially diluted and added to cell culture medium (Figure 1A).

The cytotoxicity effect was characterized by aggregation of degenerated cell clusters detaching from the bottom of the wells forming holes (gap formation) within the cell monolayer (Figure 1C). The results of cytotoxic effects of each plant extract are represented in Table 2. No effect was observed at the concentrations varying from 5 to 10 μg ml^−1^ for EO, 10 to 100 μg ml^−1^ for EE and 5 to 200 μg ml^−1^ for AE. The CC_50_ values determined by the colorimetric assay were 48.70 μg ml^−1^, 189 μg ml^−1^ and 298 μg ml^−1^ for EO, EE and AE, for concentrations up to 10, 100 and 200 μg ml^−1^ of EO, EE and AE, respectively. Cytotoxicity was detected when degenerated cells become round and float in the medium with more prominent nuclei. The EO induced the highest cytotoxic effect, while EE and AE induced moderate and low cytotoxicity effect, respectively.

### Virus inhibition

The viral CPE usually appeared within 3 days after inoculation. The CPE of BHV-1 is characterized by cell rounding and ballooning, forming grape-like clusters of spherical cells gathered around a gap in the monolayer. Sometimes, giant cells with several nuclei may be observed (Figure 1B).

**Figure 2:**
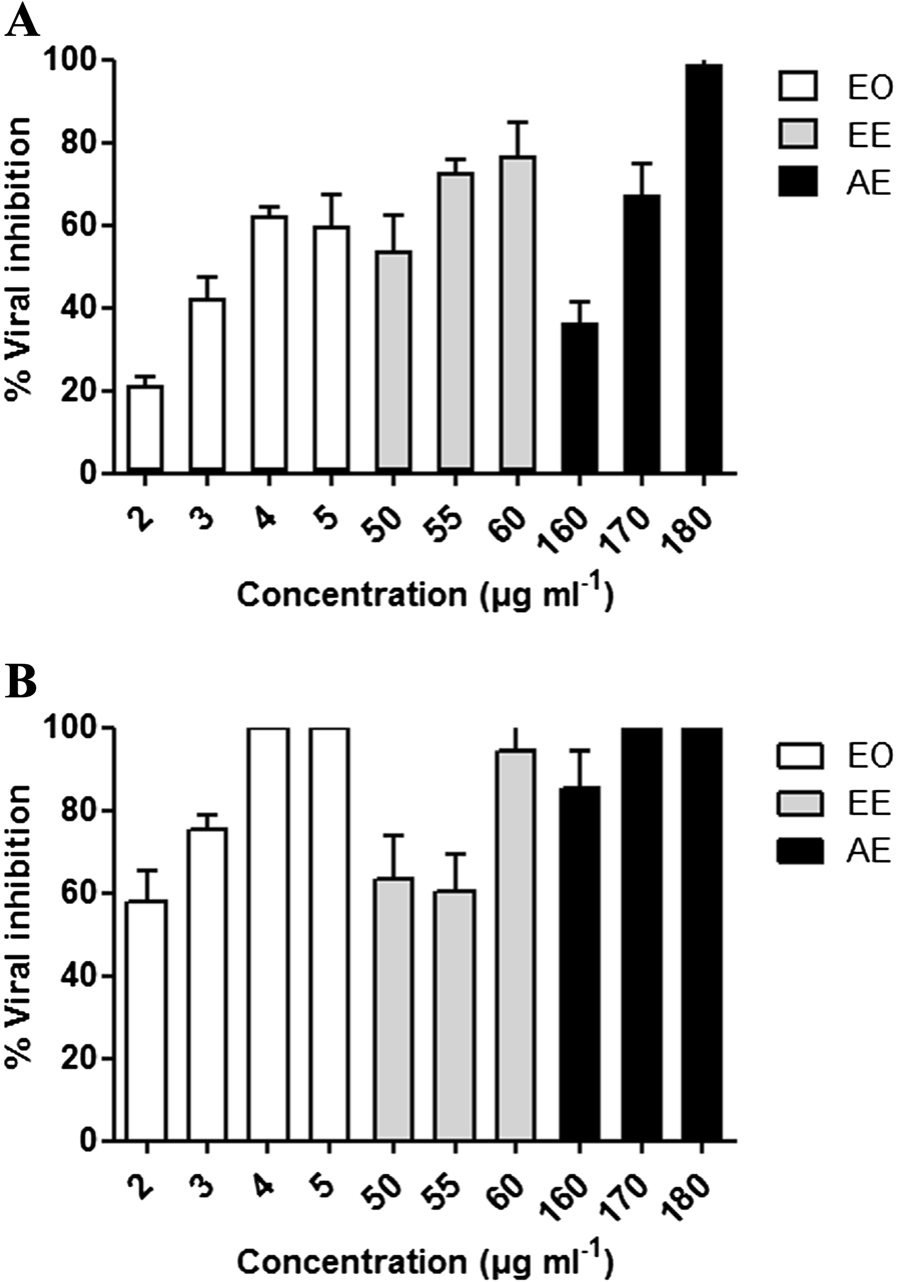
**Percentage of viral inhibition by different** ***Thymus capitata*** **extracts: Essential oil (2, 3, 4 and 5 μg ml** ^**−1**^ **), Ethanolic extract (50, 55 and 60 μg ml** ^**−1**^ **) and Aqueous extract (160, 170 and 180 μg ml** ^**−1**^ **) were tested during (A) and after (B) infection of MDBK cells with 10** ^**3**^ **TCID**_**50**_ **ml** ^**−1**^ **of Bovine herpesvirus type 1.**

At MNTC of plant extracts, viral CPE was significantly reduced (p-values < 0.05) and the anti-BHV-1 activity showed a dose dependant response (Figure 2A and B). The IC_50_ were 3.36, 47.8 and 164 μg ml^−1^ for EO, EE and AE, respectively. According to the SI values, the highest antiviral activity was seen with EO followed by that of EE and AE (Table 3). Indeed, a high cytotoxic value and a low inhibitory concentration gave the highest selectivity index for EO, indicating that it is the most effective antiviral extract (Table 3).

When host cells were treated with extracts prior to infection, no CPE of t BHV-1 infection was observed (Figure 1D). Thus, pre-treatment of MDBK cells with *T. capitata* EO, EE and AE completely inhibited BHV-1 infectivity (p-value < 0.05). However, when *T. capitata* EO, EE or AE were added during or after virus penetration, the viral CPE of the BHV-1 was significantly reduced in a dose dependant manner (p-value < 0.05) (Figure 2A; B).

A very significant correlation was found between AE concentration and viral inhibition in the interval [160, 180 μg ml^−1^] of AE concentration.

## Discussion

The analyses of the composition of EO, EE and AE of Tunisian *T. capitata* revealed the predominance of phenolic compounds (Table 1). Thymol: 3-Methyl-4-isopropylphenol was the major component found in both EO and EE. Caryophyllene was present in low concentrations in EO and EE. However, camphor, linalool, eugenol and borneol were only identified in EE and EO, respectively. Interestingly, AE exhibited the largest amount of apigenin- and luteolin as phenolic (flavones) compounds. In contrast, rosmarinic acid was found to be a minor compound in AE (Table 1). These findings are in accordance with those reported in previous studies on *T. capitata* from South Tunisia and other countries [14,19]. Mkaddem *et al.* [19] showed that the major EO compound in *T. capitata* was thymol, using plants from the same region. Also, Behravan *et al.* [14] showed that thymol and carvacrol were the major compounds in *T. capitata* EO from Iran. However, other studies showed geographic variations of chemical compositions of *T. capitata* extracts [15,20]. Nolkemper *et al.* [11] found that rosmarinic acid was the most abundant compound of the same genus *T. vulgaris* AE*.* In fact, bioactive molecule types and amount variations from *T. capitata* depend on the geographic distribution (climate, soil type), the collecting season, as well as, the plant part [20,21].

In the present study, *T. capitata* extracts exhibited an *in vitro* cytotoxic activity with CC_50_ values varying from 48.70 to 298 μg ml^−1^, depending on the type used in the same biological conditions (Table 2). When referring to the *in vitro* and the predicted *in vivo* substance toxicity classification of Halle and Göres [22]*, T. capitata* EO, EE and AE are considered to exhibit a relatively low toxicity; the most toxic extract being EO.

Cytotoxic activities of EO or its major components were demonstrated, *in vitro*, in mammalian cells to be moderate to high [22]. Indeed, EO induced some defects in the cell respiratory system (permeabilization of outer and inner mitochondrial membranes), usually directly associated with cell death by apoptosis and necrosis [23]. In general, the cytotoxic activity of EO is mostly due to the presence of phenols. However, flavones are the cytotoxic components present in AE and have been reported to exhibit cytotoxic activity at high concentrations towards normal non infected human cells [24]. Furthermore, it has been suggested that when flavonoids are introduced into cells, they increase the intracellular reactive oxygen species levels, and exert their cytotoxicity effect [24]. According to previous studies, CC_50_ values of *T. vulgaris* were 70, 62.3 [12] and 62 μg ml^−1^ [11] for EO, EE and AE, respectively. The difference in AE chemical compositions between *T. capitata* and *T. vulgaris* may explain the observed CC_50_ values. The rosmarinic acid was the most abundant compound in *T. vulgaris*. However, for South Tunisia, *T. capitata* AE, apigenin and luteolin were the major compounds and rosmarinic acid was the minor extracted compound. Nolkemper *et al.* [11] showed that for aqueous *Lamiaceae* plant extracts, the CC_50_ varied between 55 and 125 μg ml^−1^ due to differences in their phenolic compounds’ concentrations.

In the present study, the inhibitory effect of thyme EO against BHV-1 infection was compared to the antiviral activity of EE and AE (Table 3). Both *T. capitata* extracts were shown to affect significantly BHV-1 replication (p value < 0.05). Previous *in vitro* experiments showed similar results for EO, EE and AE from *Lamiaceae* plant including *Thymus* spp*.* [11,12].

The thyme EO revealed a higher antiviral activity and a SI of 14.49, whereas EE and AE showed lower SIs (Table 3). The anti-herpes virus activities of several EOs of different plant sources as well as of some constituents of EOs were reported previously, e.g. peppermint oil, thyme oil and anise oil [10,25,26]. The presence of thymol and β-caryophyllene in EO may strongly contribute to their antiviral effect which is in agreement with our results and as described by others [14,27].

Pre-treatment of cells with different *T. capitata* extracts, at the tested concentrations, completely inhibited viral CPE (Figure 1D). In addition, an antiviral activity dose dependent effect was significantly observed (p-value < 0.05) for EO, EE and AE when added during or after cell infection (Figure 2A; B). A very significant correlation was found between AE concentration and viral inhibition in the interval [160, 180 μg ml^−1^]. These results suggested that these biomolecules may interfere with either viral particles and/or virus envelope, and may mask viral structures or cell receptors necessary for the virus adsorption and entry into the host cells. In fact, Schnitzler *et al.* [13] reported that balm oil, abundant in phenolic compounds, affected the viruses *in vitro* when added before viral adsorption, although the mechanism of action is still unknown. It is suggested that the balm oil could bind to the viral proteins involved in the host adsorption and penetration, or damage the virus envelope. In several cases, plant-derived polyphenols exhibit anti-herpesvirus activity mostly by influencing the early phases of infection [18]. Furthermore, Reichling *et al.* [12] concluded that ethanolic plant extracts affect herpesvirus prior to and during adsorption. They suggested that the antiviral activity is due to the association of the extracts with proteins of the host cell surface molecules, resulting in reduction or prevention of viral adsorption.

Flavonoid derivatives have also been reported to possess antiviral activity against a wide range of viruses [28–30]. Apigenin 7-O-glucoside and luteolin are flavonoid compounds that are found in lemon balm (*Melissa officinalis*), chamomile (*Chamomilla recutita*), and celery (*Apium graveolens*) and have shown antiviral activity *in vitro* [28,31]. The extracts of basil (*Ocimum basilicum*) and their purified compounds, including apigenin, were tested against a number of viruses (herpesvirus, adenovirus, and hepatitis B virus), and have displayed a broad range of antiviral action, which is in accordance with our data [32].

The mechanism of antiviral action of polyphenolic compounds is mainly due to inhibition of viral enzymes, disruption of cell receptors, prevention of viral binding and penetration into cells. Finally, some natural compounds are reported to have the capacity to interfere with herpesvirus enzymes (DNA polymerase inhibition by eugenol), and thus viral replication [33].

## Conclusion

*Thymus capitata* EO, EE and AE affect (below cytotoxic concentration) mainly the adsorption of BHV-1 virus to Madin-Darby Bovine Kidney host cells. This activity can be explained by the presence of some flavonoids such as apigenin, luteolin or rosmarinic acid in the AE and the presence of polyphenols as thymol in EE and EO. During pre-treatment of MDBK cells, all tested extracts of *T. capitata* inhibit viral attachment to cell receptors making them good candidates against viral infection. Moreover, a dose-dependent effect was recorded during and after viral infections for the three *T. capitata* extracts tested.

## Abbreviations

GC/MS: Gas chromatography–mass spectrometry
HPLC/MS: Performance liquid chromatography/mass spectrometry
EO: Essential oil
EE: Ethanolic extract
AE: Aqueous extract
BHV-1: Bovine herpesvirus type 1
DMEM: Dulbecco’s modified Eagle’s medium
DMSO: Dimethylsulfoxide
MNTC: Maximum non-toxic concentration
MDBK: Madin-Darby Bovine Kidney
CPE: Cytopathic effect
TCID_50_: The 50% Tissue Culture Infective Dose
IC_50_: The 50% effective antiviral concentration
CC_50_: The 50% cytotoxic concentration
SI: The selectivity index
